# The Western Lancet and Hospital Reporter

**Published:** 1850-08

**Authors:** 


THE WESTERN LANCET AND HOSPITAL REPORTER.

We are pleased to see that Dr. Geo. Mendenhall, of Cincinnati, has become one of the editors of the Western Lancet. He stands deservedly high in the estimation of the community in which he resides, and we cordially welcome him to the editorial ranks, and wish him a prosperous career. B.

